# Searching for Cutaneous Leishmaniasis in Tribals from Kerala, India

**DOI:** 10.4103/0974-777X.62874

**Published:** 2010

**Authors:** Simi S M, Anish T S, Jyothi R, Vijayakumar K, Rekha Rachel Philip, Nimmy Paul

**Affiliations:** *Department of Dermatology and Venereology, Government Medical College, Thiruvananthapuram, India*; 1 *Department of Community Medicine, Government Medical College, Thiruvananthapuram, India*; 2 *Department of Microbiology, Government Medical College, Thiruvananthapuram, India*

**Keywords:** Cutaneous leishmaniasis, India, kerala, Tribal

## Abstract

**Background:**

In India, indigenous cases of cutaneous leishmaniasis (CL) are mainly confined to the northwestern region. But now, more and more case reports are coming in from other parts of India. In January 2009, a 26-year-old lady residing in a forest area in Thiruvananthapuram district of Kerala State presented with bluish red nodules on her upper extremities, of six months duration, which was clinically more in favor of cutaneous leishmaniasis. She had never gone out of the district of Thiruvananthapuram in her life.

**Aim:**

To investigate whether the patient hails from a new endemic focus of cutaneous leishmaniasis.

**Setting and Design:**

An epidemiological investigation in the form of a survey was carried out in March 2009 by a multidisciplinary team among 63 persons residing in the Mele Aamala and Aayiramkala forest tribal settlements in Kuttichal Panchayat of Thiruvananthapuram district.

**Material and Methods:**

History taking and clinical examination of 38 persons in the area with special consideration to skin lesions was undertaken. Microbiological and histopathological examination of the skin lesions was done. Breeding places of sand fly and possible reservoirs of *Leishmania* were also simultaneously investigated.

**Statistical analysis used:**

The data obtained was tabulated as frequency and percentage. Chi-square test was done to find out the statistical significance of differences in distributions.

**Results:**

Out of the 38 persons examined, active lesions were found in 12 persons and six had healed lesions. Tissue samples were obtained from seven out of the 12 suspected cases. Four of them showed Leishman Donovan (LD) bodies in tissue smears. Out of the cultures taken from three patients, one showed promastigote forms in Novy McNeal Nicolle (NNN) medium. Histopathological study was done in five patients and two patients had LD bodies, one had epithelioid cell granuloma and the other two had mixed infiltrate with predominantly macrophages. All the three investigations were carried out in three patients and out of them one showed positivity in all the three investigations and the rest two were positive in tissue smear and histopathological examination. Sandflies collected from the area gave an indirect evidence of its role in the disease transmission in the area.

**Conclusion:**

The clinical, microbiological and histopathological evaluation of the skin lesions was consistent with cutaneous leishmaniasis. But none of the patients gave history of travel outside the district before the onset of the disease and no one had newly moved into this area within the last two years. So this may be considered as probably a new focus of cutaneous leishmaniasis

## INTRODUCTION

Leishmaniasis is endemic in 88 countries throughout Africa, Asia, Europe, and North and South America. There are an estimated 12 million cases worldwide, with 1.5 to 2 million new cases each year.[1]

The sixtieth World Health Assembly, in its resolution, recognized leishmaniasis as one of the most neglected tropical diseases.[2] In India, indigenous cases of cutaneous leishmaniasis (CL)- anthroponotic cutaneous leishmaniasis (ACL) as well as zoonotic cutaneous leishmaniasis (ZCL) - are mainly confined to the hot dry northwestern region and are endemic in the western Thar Desert of Rajasthan.[3] Two cases of CL were reported in Kerala, India, for the first time in 1988 in Thiruvananthapuram, imported from Saudi Arabia.[4] The first indigenous case in the State was reported from Malappuram district after two years.[5] Suspected cases of CL were reported from Kollam district of Kerala during 2001- 2003.[3]

**Figure 1:**
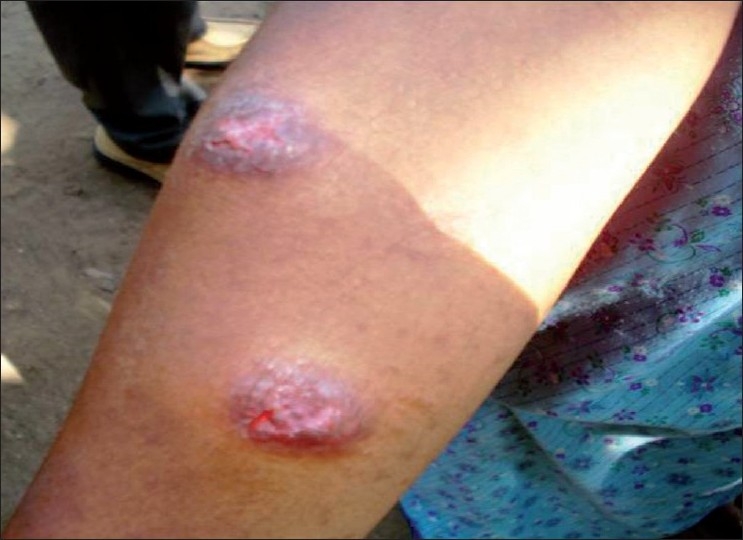
Violaceous nodules over right forearm

In January 2009, a 26-year-old female patient came to the Out Patient (OP) clinic of the Department of Dermatology and Venereology, Government Medical college, Thiruvananthapuram, Kerala with a six month history of mildly itchy reddish raised lesions over her upper extremities. The lesions were enlarging in size, attained a bluish hue and some of the lesions developed central ulceration, which prompted her to seek expert medical care. She resided in a forest area in the tribal settlement of Mele Aamala in Kuttichal area of Thiruvananthapuram district, Kerala. On examination, there were six well defined erythematous indurated nodules with violaceous hue varying in size from about 2 cm to about 6 cm over both forearms predominantly over the extensor aspect [Figure 1]. The patient did not give any history of travel outside the district. Presence of nodular lesions on the exposed parts of the body with no sensory loss, the central ulceration of the nodules and residence in a forest area prompted us to suspect CL. A skin biopsy from the edge of a nodule was done, which showed a granulomatous reaction with epithelioid cell granuloma with Langhan's type of giant cells. A dense lymphocytic infiltrate and focal necrosis was also seen. Special stain for Acid Fast Bacilli (AFB) and Leishman Donovan (LD) bodies (Giemsa) were negative in the histopathological section.

The patient revealed that her parents, daughter and a few other persons in her neighborhood also had similar lesions. This prompted us to set up a multidisciplinary team including dermatologists, epidemiologists, entomologists, microbiologists, pathologists and veterinary pathologists to investigate the clustering of skin lesions.

## MATERIAL AND METHODS

The study was conducted at the Mele Aamala and Aayiramkala forest tribal settlements in Ward 5 of Kuttichal Panchayat of Thiruvananthapuram District in March 2009. It is about 18 kilometers away from the Kuttichal Primary Health Centre. The index patient was from the Mele Aamala settlement. These two settlements together have 16 families with a population of 63. Twenty four of them live in the Mele Aamala settlement and 39 in Aayiramkala. The main occupation of the people there is collection of honey, rubber tapping, and farming.

The inhabitants resided in a difficult-to-reach area in which the houses were far apart in the forest. So we attempted to mobilize people with skin complaints with the help of community leaders and volunteers. A thorough history taking and clinical examination of the inhabitants with special emphasis on skin lesions was undertaken. A total of 38 persons in the locality were examined. The details were noted in a structured proforma. It was decided to carry out invasive procedures only on adult patients who agreed for the same. A tissue smear (slit skin smear) was taken from the edge of the lesions. From each patient, three tissue smears were taken. Lesions and the adjacent normal-looking skin around them were cleaned and sterilized with disinfectant. Tissue for making stained smears was taken using a disposable scalpel blade (no. 11). A small incision was made in the cleaned margin of the skin lesions with the point of the blade. The blade was turned 90 degrees and scraped along the cut edge of the incision to remove and pick up skin tissue, which was smeared on a clean glass microscope slide. After smears had dried completely, they were fixed with 100% methanol, allowed to dry again, and stained with leishman's stain for microscopic examination. Skin biopsy specimens were taken for histopathological examination from typical lesions in adult patients. Because of the field restrictions it was decided to collect specimens for culture and histopathological examination from only a limited number of patients. Histopathological specimens were subjected to special stain for Acid Fast Bacilli and Giemsa stain for Leishman Donovan (LD) bodies. Sterile saline (0.1 to 0.2 ml) was drawn into a syringe, and the needle was inserted into the nodule or ulcer's margin and rotated gently several times. A small amount of saline was expressed into the tissue, the needle was rotated, and some tissue aspirate and freed tissue were withdrawn. The syringe was removed from the lesion, and some of its contents were expressed into tubes containing semisolid rabbit blood agar medium -Novy McNeal Nicolle medium (NNN medium). An epidemiological investigation with special emphasis on the breeding places of sand fly and possible reservoirs was also simultaneously conducted. A veterinary pathology team collected blood samples and lymph node aspirates from four domestic dogs and blood samples from 10 poultry from the households of the patients with active lesions. Sand flies were also collected from the region using two methods. Castor oil soaked papers were placed to collect sand flies at a height of one to five feet from the ground level in the mud walls of the houses and tree crevices. Some sand flies were collected directly in to the test tubes after locating them on the domestic animals.

Using a clinical criteria devised by Bari and Rahman, a diagnosis of CL was made if the patient met at least five of the following eight clinical criteria: (1) origin from a known endemic area or a history of having visited these areas in the past six months, (2) discrete nodules or non healing ulcers, (3) largely painless and non-itchy lesions, (4) few lesions, (5) duration of several days or weeks, (6) resistant to conventional antibiotics, (7) history of similar lesions in the household or locality, (8) some additional morphological patterns, if present, for example, satellite papules, subcutaneous nodules with lymphatic spread (sporotrichoid pattern), paired or clustered lesions or volcanic nodules.[6] Information on age, gender, and duration of disease, type, site, number and nature of the skin lesions, treatment taken and history of travel during the last one year were also collected.

### Ethics

Tissue samples were not collected from children, elderly and those suffering from any debilitating illness. Written consent was obtained from patients before collecting tissue specimen. The consent for those less than 18 years was obtained from their parents. The clinical examination and specimen collection was done in the presence of the local Panchayat representative and Panchayat President. The collection of specimen from the domestic animals (dogs and poultry only) and the search for sand flies in the domestic and peridomestic area was conducted with the consent of the head of the families.

### Statistical analysis

The data obtained was tabulated as frequency and percentage. Chi-square test was done to find out the statistical significance of differences in distributions.

## RESULTS

Out of the 38 persons examined, active lesions were found in 12 persons and six had healed lesions. Of the 12 patients with active skin lesions, six (50%) were women. The age varied from one to 41 years. The mean age (Standard Deviation) was 18.67(13.2) years. Among the patients, there were three each of home makers and students, two each of manual workers, forest produce gatherers and pre -school children. All the patients were living in houses made of mud, cement, bamboo and coconut leaves. Only three patients had a history of travel outside the State, ever in their lifetime, to Pondichery and Andhra Pradesh (Puttaparthy), which are non endemic areas of CL. Moreover, all of them had skin lesions even before their travel. No one had moved into this area within the last two years.

Duration of illness varied from two months to one year with a mean duration of 5.75 months (SD 3.6). The lesions were present on the face and upper limbs in females; and face, upper limb and upper part of trunk in males, which are the usual exposed parts in females and males respectively. Out of the 12 patients, 11 had lesions on exposed parts. The exception was a seven year old girl who had an erythematous papule on her gluteal region. The number of lesions varied from one to ten with a mean of 3.92 (SD 2.3). The younger patients (age ≤ 14 years) had significantly lower number of skin lesions compared to adults [Table 1].

**Table 1:** Distribution of number of active skin lesions of patients with age

Age group	Number of active skin lesions (Mean)
Age ≤14 years (n=5)	2.17
Age >14 years (n=7)	5.67

t = 4.27, df = 10, *P* = 0.002

**Figure 2:**
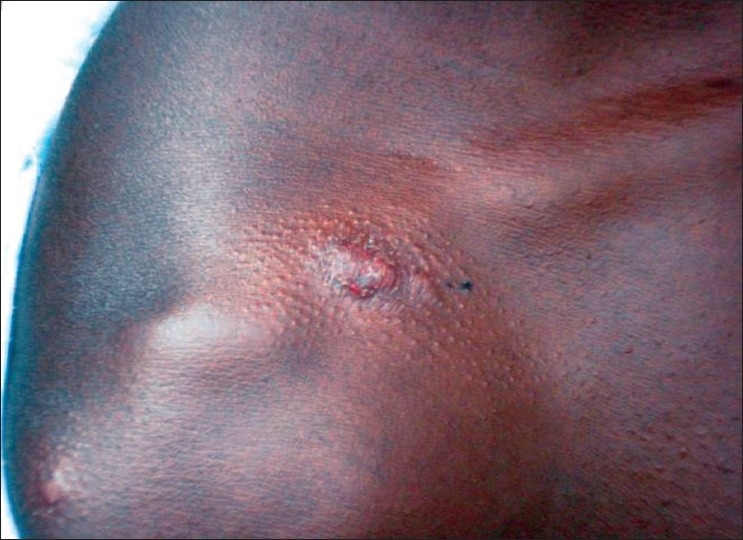
Erythematous papules over right shoulder and back of trunk

The type of lesions included nodules, nodulo-ulcerative lesions, papules, plaques and healed scars. The majority of lesions were erythematous papules [Figure 2]. Some of the lesions were bluish red or skin colored. Mucosal involvement in the form of an erythematous papule was seen on the upper lip of one patient. The details of the clinical presentation of the lesions are given in Table 2. None of them had sensory loss over the lesions. Six persons had past history of erythematous papules on the exposed parts within the last 18 months which healed to form scars. Five of them were males. Age varied from 11 to 105 years. Two of them had a few hypopigmented skin lesions.

**Table 2:** Distribution of the features of lesions among patients with active skin lesions

Clinical feature	Number of patients (n=12)	Percentage
Pain (mild)	2	16.7
Itching (mild)	5	41.7
Ulceration	3	25
Clustering	5	41.7
Spontaneous healing	5	41.7

**Figure 3:**
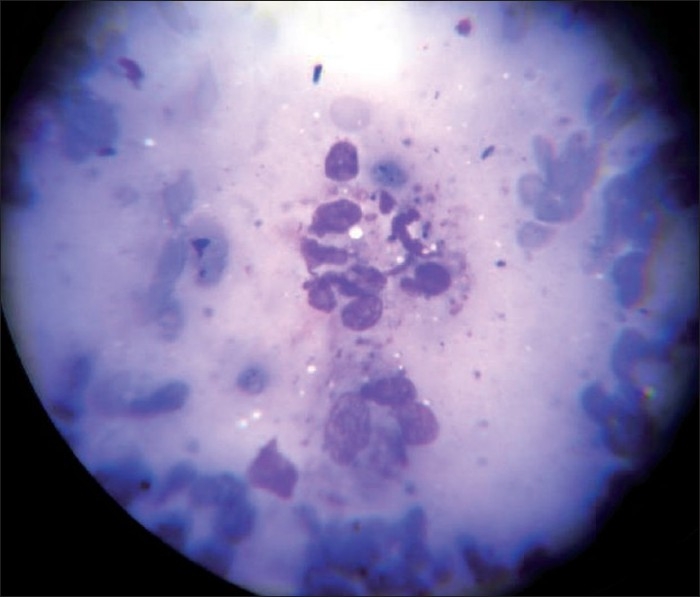
Leishman's stain of tissue smear showing LD bodies (magnification 400×)

Nine (75%) patients had at least one more person with similar illness sharing the same roof. Three of the patients had taken treatment in the form of topical or systemic antibiotics. Even multiple courses of antibiotics were not of much relief. Tissue smears were obtained from only seven out of the 12 patients. The rest were children. Out of the seven patients from whom tissue smears were taken, four showed LD bodies [Figure 3]. Skin biopsy specimens for histopathological examination were collected from five out of the seven adult patients with active lesions. The rest of the two patients were not willing for the procedure. Histopathological examination showed granulomatous lesion with basophilic bodies compatible with CL in two patients. These patients had LD bodies in their tissue smears also. One of the three patients in whom no LD bodies were demonstrated in the skin biopsy showed epithelioid granuloma with Langhan's giant cells and focal necrosis and two of them showed mixed dermal infiltrate with predominant macrophages. Special stain for AFB was negative in all.

**Figure 4:**
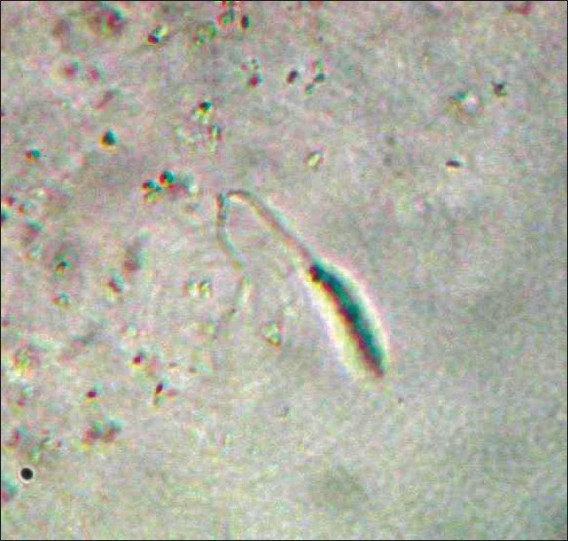
Promastigote in culture in NNN medium (magnification 100×)

**Figure 5:**
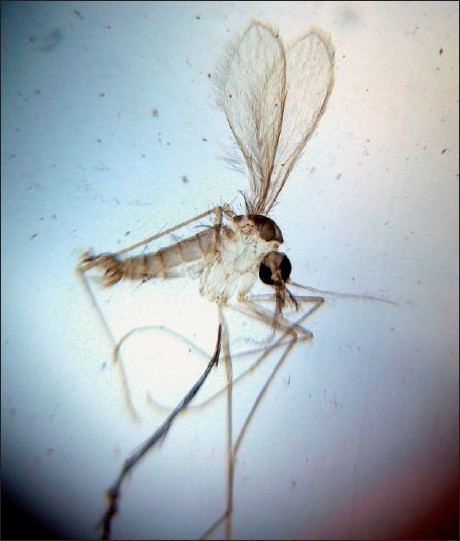
Magnified view of Sand fly collected from around the houses

Since we had only very few culture bottles at our disposal and also because of the field restrictions, specimen for culture was obtained from only three adult patients who had early active lesions. Culture done in NNN medium yielded motile promastigotes (flagellate form) in the specimen obtained from one patient within seven days [Figure 4]. All the three investigations were carried out in three patients and out of them one showed positivity in all the three investigations and the rest two were positive in tissue smear and histopathological examination. No LD body could be demonstrated in the blood samples or lymph node aspirates obtained from dogs or blood samples from poultry. We could collect sand flies from around the houses [Figure 5]. The species of sand fly has been identified as *Phlebotomus argentipes* by scientists from the National Institute of Communicable Diseases, Kozhikode, Kerala, India.

The seven adult patients in whom the investigations were carried out and the five children with active skin lesions were brought to the Department of Dermatology Medical College, Thiruvananthapuram and a thorough clinical systemic examination as well as ultrasonography of abdomen, chest X-ray, complete blood count and biochemistry panel were done and visceral Leishmaniasis was ruled out. The four patients in whom any laboratory evidence of CL was obtained were administered oral fluconazole 200 milligram twice daily for six weeks along with topical ketoconazole. The remaining eight patients were given only topical ketoconazole. The choice of the treatment was based on availability, better side effect profile and the relatively milder disease pattern. The patients underwent regular follow-up. It was found that the papules had disappeared and the nodules decreased in size. The patients are still under follow-up.

## DISCUSSION

Even though there was equal gender distribution among the patients, there was a difference in the distribution of skin lesions. In addition to face and upper-limbs, trunk was also predominantly involved in males. This pattern has been described in case of vector borne diseases elsewhere and can be considered as an indicator of a vector borne disease. This difference in the pattern of skin lesions could be explained with their clothing patterns. Almost all age groups were affected but the number of skin lesions was significantly less in the younger age group. A study done in a large sample of CL patients in Kabul also points out that elderly people are at slightly greater risk of having active lesions.[7] It may be due to some immunological characteristics and further studies are needed in this regard.

In a study done by Centers for Disease Control in 2002 – 2003 among U.S military personnel who developed Cutaneous Leishmaniasis, it was seen that the patients had a median of three (range: one to nine) skin lesions, which ranged from 3 mm to 40 mm in diameter. Higher proportions of the lesions were located on the upper (39%) or lower (32%) extremities than on the trunk/back (16%) or face/neck (13%). These findings are similar to the findings seen in our study.[8] The typical lesion of CL is a skin nodule.[8] But in our study only one patient had nodule and majority had erythematous papules. But it is reported that lesions like erythematous papules can occur in CL.[9] Mucosal involvement in the form of upper lip lesion was seen in one patient. Mucosal lesions are rarely caused by *Leishmania tropica* and they occur usually on or around muco cutaneous junctions such as lips or nostrils.[10]

Yield of LD bodies in our study is high (4/7 i.e, 57.14%), may be due to the comparatively newer lesions sampled.[11] Previous report of CL from Kerala had only 2/11(18.18%) clinically diagnosed cases positive for LD bodies.[12] LD bodies were obtained in two histopathological specimens. As a rule in CL, organisms are scarce and difficult to identify in sections stained with Hematoxylin and Eosin stain.[13] The presence of LD bodies in tissue smears and failure to demonstrate them in histopathology sections remains unexplained. Bahamdam *et al*. made similar observations in three of their 21 patients and postulated that probably *Leishmania* organisms appear larger in touch smears and are scanty in histopathology due to repeated processing of biopsy specimens with dehydrating solutions.[14] Histopathological examination results were consistent with Types V,IV and III of Ridley's histopathological classification of CL.[15] Efforts are on to identify all the patients in this difficult-to-reach area and to effectively contain this probably new focus of CL.

The identification of the sand fly species as *Phlebotomus argentipes* may be considered as an indirect evidence of the causative organism being *LD*. But our attempts to isolate Leishmania species using Polymerase Chain Reaction failed. Even though *Leishmania donovani* is more associated with visceral Leishmaniasis, scientists have isolated *Leishmania donovani* as a predominant pathogen among patients with CL in a new focus in Himachal Pradesh, India.[10]

### Limitations of the study

The study setting was a difficult-to-reach area and so all the persons in the locality could not be examined and investigated. Attempts to identify an animal reservoir were minimal. We could not succeed in the identification of the species of Leishmania.

## CONCLUSION

The clinical, microbiological and histopathological evaluation of the skin lesions was consistent with Cutaneous Leishmaniasis. None of the patients gave history of travel outside the district before the onset of the disease and no one had newly moved into this area within the last two years. So this may be considered as an indirect evidence of a new indigenous focus of CL. Newer diagnostic modalities like polymerase chain reaction may help in species identification of Leishmania and there by confirming the presence of a new endemic focus of CL.

This is a first report of CL from Thiruvananthapuram district, the State head quarters. The recognition of this probably new focus of CL in a tribal area deserves special attention. Tribals are often a marginalized section of the society. Little attention is given towards their needs by the policy makers. To make the situation worse, they are not empowered enough to voice their needs. So it is desirable that public health authorities make every effort to contain this new focus.
